# Maternal PM2.5 exposure is associated with preterm birth and gestational diabetes mellitus, and mitochondrial OXPHOS dysfunction in cord blood

**DOI:** 10.1007/s11356-023-31774-0

**Published:** 2024-01-10

**Authors:** Young-Ah You, Sunwha Park, Eunjin Kwon, Ye-Ah Kim, Young Min Hur, Ga In Lee, Soo Min Kim, Jeong Min Song, Man S. Kim, Young Ju Kim, The APPO study group, Young-Han Kim, Sung Hun Na, Mi Hye Park, Jin-Gon Bae, Geum Joon Cho, Soo-Jeong Lee

**Affiliations:** 1 Department of Obstetrics and Gynecology and Ewha Medical Institute, College of Medicine, Ewha Womans University, 1071, Anyangcheon-Ro, Yangcheon-Gu, Seoul, 07985 Republic of Korea; 2 Division of Allergy and Respiratory Disease Research, Department of Chronic Disease Convergence, National Institute of Health, Cheongju, 28159 Republic of Korea; 3 Translational-Transdisciplinary Research Center, Clinical Research Institute, Kyung Hee University Hospital at Gangdong, College of Medicine, Kyung Hee University, Seoul, Republic of Korea; 4 Department of Biomedical Science and Technology, Graduate School, Kyung Hee University, Seoul, Republic of Korea; 5 Department of Obstetrics and Gynecology, Kyung Hee University Hospital at Gangdong, Kyung Hee University, Seoul, Republic of Korea

**Keywords:** Fine particulate matter, Pregnancy complications, Mitochondria, Oxidative phosphorylation, Cord blood, Preterm birth, Gestational diabetes mellitus

## Abstract

Maternal exposure to fine particulate matter (PM_2.5_) is associated with adverse pregnancy and neonatal health outcomes. To explore the mechanism, we performed mRNA sequencing of neonatal cord blood. From an ongoing prospective cohort, Air Pollution on Pregnancy Outcome (APPO) study, 454 pregnant women from six centers between January 2021 and June 2022 were recruited. Individual PM_2.5_ exposure was calculated using a time-weighted average model. In the APPO study, age-matched cord blood samples from the High PM_2.5_ (˃15 ug/m^3^; *n* = 10) and Low PM_2.5_ (≤ 15 ug/m^3^; *n* = 30) groups were randomly selected for mRNA sequencing. After selecting genes with differential expression in the two groups (*p*-value < 0.05 and log2 fold change > 1.5), pathway enrichment analysis was performed, and the mitochondrial pathway was analyzed using MitoCarta3.0. The risk of preterm birth (PTB) increased with every 5 µg/m^3^ increase of PM_2.5_ in the second trimester (odds ratio 1.391, *p* = 0.019) after adjusting for confounding variables. The risk of gestational diabetes mellitus (GDM) increased in the second (odds ratio 1.238, *p* = 0.041) and third trimester (odds ratio 1.290, *p* = 0.029), and entire pregnancy (odds ratio 1.295, *p* = 0.029). The mRNA-sequencing of cord blood showed that genes related to mitochondrial activity (FAM210B, KRT1, FOXO4, TRIM58, and FBXO7) and PTB-related genes (ADIPOR1, YBX1, OPTN, NFkB1, HBG2) were upregulated in the High PM_2.5_ group. In addition, exposure to high PM_2.5_ affected mitochondrial oxidative phosphorylation (OXPHOS) and proteins in the electron transport chain, a subunit of OXPHOS. These results suggest that exposure to high PM_2.5_ during pregnancy may increase the risk of PTB and GDM, and dysregulate PTB-related genes. Alterations in mitochondrial OXPHOS by high PM_2.5_ exposure may occur not only in preterm infants but also in normal newborns. Further studies with larger sample sizes are required.

**Supplementary Information:**

The online version contains supplementary material available at 10.1007/s11356-023-31774-0.

## Introduction

Fine particulate matter (PM_2.5_) is the main contributor to air pollution and is the fourth leading risk factor for death and disability worldwide (Anderson et al. 2012). PM_2.5_ can penetrate deeply into the lungs and circulate through the bloodstream, causing serious health effects, including cardiac arrhythmia, coronary heart disease, and premature death (Brook et al. 2010). These particles can also pass through the maternal–fetal placental barrier (Bové et al. 2019; Wick et al. 2010), which can adversely affect mothers and newborns, resulting in preterm birth, elevated blood pressure, gestational diabetes mellitus, and low birth weight (Shah and Balkhair 2011; Zhang et al. 2018; Madhloum et al 2019). In particular, according to the Developmental Origins of Health and Diseases theory, prenatal exposure to PM_2.5_ can have lifelong consequences because perturbations in the intrauterine environment are involved in the development of disease in later life (Barker 1990).

The mechanisms underlying PM-induced health effects include increased oxidative stress and inflammation (Kelly 2003; Schins et al. 2004). Various components of PM, including environmentally persistent radicals, peroxides, aromatic compounds, and dissolved metals, can generate reactive oxygen species (ROS), leading to oxidative stress and consequently enhancing various biological processes, such as inflammation and cell death (Jiang et al. 2019; Kamdar et al. 2008; Møller et al. 2014). Mitochondria are major locations for ROS production and cellular targets for the damaging effects of PM (Fetterman et al. 2017; Jin et al. 2018). ROS generation and the subsequent increase in oxidative stress have been recognized as a major contributor to cell damage, cell death, DNA damage, and inflammation due to PM_2.5_ exposure (Kamdar et al. 2008; Li, et al. 2003). As ROS are mainly produced in the mitochondria as by-products of cellular respiration (Ward 2017), the disruption of mitochondrial electron transport (oxidative phosphorylation, OXPHOS) can further augment ROS production and amplify oxidative stress (Ward 2017). However, studies on the specific connections between PM_2.5_ exposure and mitochondria in a prospective cohort study are limited.

The regulation of gene expression is fundamental for linking genotypes to phenotypes. The synthesis and maturation of RNAs are tightly controlled and form complex gene expression networks that ultimately drive biological processes (Marguerat and Bähler 2010). High-throughput mRNA sequencing (mRNA-seq) can derive millions of nucleotide sequences from individual transcripts (Stark et al. 2019). These nucleotide sequences provide multiple coverages of the entire transcriptome. RNA-seq is widely used in the study of diseases and biological processes because it can identify genes that are actively transcribed in a sample and quantify the level at which alternative transcripts of a gene are transcribed (Ura et al. 2022).

In this study, we report the association between adverse pregnancy outcomes and PM_2.5_ exposure in pregnant women recruited from the ongoing prospective cohort, Air Pollution on Pregnancy Outcome (APPO) study. We report on the genes associated with preterm birth (PTB) and mitochondrial dysfunction associated with oxidative stress and inflammation using mRNA-seq of cord blood.

## Methods

### Study population

In the Air Pollution on Pregnancy Outcome (APPO) study, an ongoing prospective cohort, 454 pregnant women (all singleton) were recruited for this study by the APPO study group at six centers between January 2021 and June 2022. Details of the APPO cohort study were previously reported (Hur et al. 2023). The hospitals were located in a metropolitan area, an industrial complex, or a mountainous area. Pregnant women were > 19 years of age before 28 weeks gestation without underlying diseases. Participating mothers completed study questionnaires during early- or mid-term pregnancy to provide detailed information on maternal age, pre-pregnancy body mass index (BMI), maternal education, occupation, smoking status, alcohol consumption, and use of medication. We collected clinical data on obstetric history, ultrasound sonography, and routine blood tests, including white blood cell counts and high-sensitivity C-reactive protein (hs-CRP) levels as inflammatory markers. After delivery, pregnancy outcomes were recorded, including gestational age at delivery, delivery mode, neonatal sex, birth weight, birth height, APGAR score, and neonatal intensive care unit admission. Blood (5 ml) and urine (15 ml) samples were collected during each trimester of pregnancy, and cord blood (5 ml) was collected at delivery. After sample collection, urine was stored at − 80 ℃ within 30 min, blood was centrifuged, and plasma and buffy coat were stored at − 80 ℃.

This study was approved by the Ethical Research Committees of the six centers (Ewha Womans University Mokdong Hospital, EUMC 2021–04-032; Ewha Womans University Seoul Hospital, 2021–04-022; Yonsei University Severance Hospital, 4–2021-0414; Kangwon National University Hospital, KNUH-B-2021–04-012–008; Keimyung University Dongsan Medical Center, 2021–04-073; and Korea University Guro Hospital, 2021GR0233) and conducted according to the ethical principles of the Helsinki Declaration. All the participants provided written informed consent.

### PM_2.5_ exposure assessment

Daily outdoor PM_2.5_ concentrations were collected from a nearby urban atmospheric measurement network based on the residential addresses of the study subjects. The Urban Air Monitoring Station data used in this study were obtained from Air Korea (https://www.airkorea.or.kr/web) of The Korean Ministry of the Environment.

The household indoor PM_2.5_ concentrations were measured by AirguardK® (Kweather, Co., Korea), a small electronic device with a light-scattering laser photometer sensor that can detect air pollution levels. The device was placed in the participants’ homes for at least 1 week during each trimester of pregnancy to measure the household indoor air quality. The measured indoor PM_2.5_ data were transmitted to the indoor air quality monitoring platform over a long-term evolution communication network to prevent data loss and to collect and store data per minute.

We calculated individual PM_2.5_ exposure using a time-weighted average model that considers the duration and location of various activities, using collected outdoor and indoor PM_2.5_ concentrations, and time-activity patterns of pregnant women (Edwards et al. 2001). The equation is shown below.

$${C}_{ind} = \left\{({C}_{household\;indoor} \times {T}_{household\;indoor}) + ({C}_{indoor\mathrm{\;not\;at\;home\;}}\times {T}_{indoor\mathrm{ \;not\;at\;home\;}}) + ({C}_{outdoor}\times {T}_{outdoor})\right\} \div 24$$

where,

*C*_ind_: individual PM_2.5_ exposure
*C*_*household*__*indoor*_: household indoor PM_2.5_ concentration
*T*_*household*__*indoor*_: time spent indoors at home
*C*_*indoor*__not at home_: average of household indoor PM_2.5_ concentration of all participants
*T*_*indoor*__not at home_: time spent indoors not at home
*C*_*outdoor*_: outdoor PM_2.5_ concentration based on address
*T*_*outdoor*_: time spent outdoors

### mRNA sequencing in cord blood

**Fig. 1:**
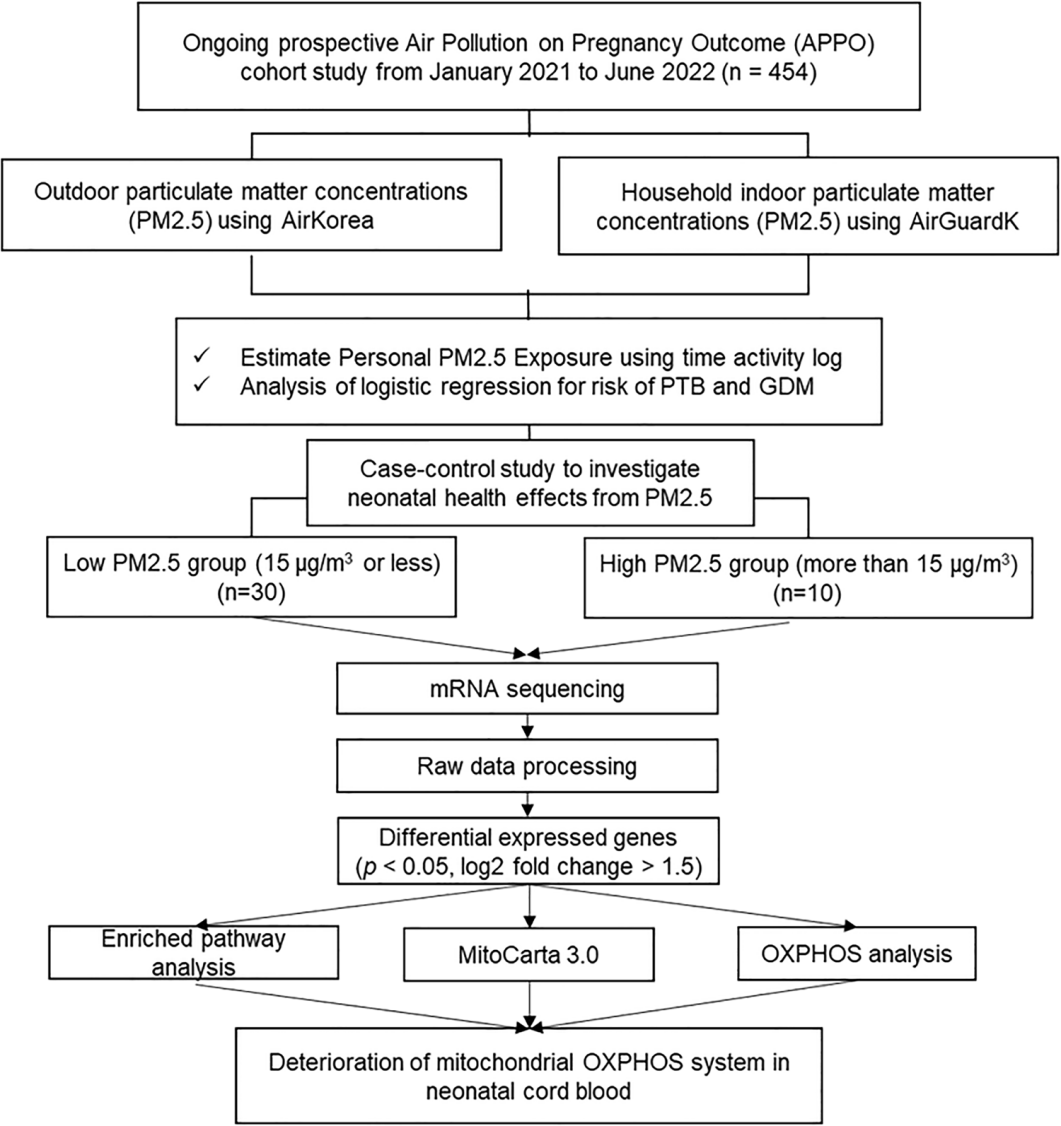
Study flow chart. PTB, preterm birth; GDM, gestatonal diabetes mellitus; OXPHOS, oxidative phosphorylation

To perform mRNA sequencing on cord blood, we categorized the participants into two groups based on individual PM_2.5_ exposure during pregnancy:15 µg/m^3^ or less for the low PM_2.5_ exposure (Low PM_2.5_) and 15 µg/m^3^ or more for high PM_2.5_ exposure (High PM_2.5_) groups (Fig. 1). This concentration followed the recommended level of 24-h average PM2.5 according to the WHO 2021 air quality guidelines. We randomly selected extracted total RNA from the buffy coats of cord blood samples from Low PM_2.5_ (*n* = 30) and High PM_2.5_ (*n* = 10) groups using the Qiagen RNA extraction kit. Following quality assessment using an Agilent Tapestation 4200 (Agilent Technologies, Santa Clara, CA, USA), RNA was subjected to poly (A) enrichment using the NEBNext Poly(A) mRNA Magnetic Isolation Module and cDNA library generation with the xGen Broad-Range RNA Library Prep kit using xGen Normalase UDI primers (Integrated DNA Technologies, Coralville, IA, USA). Subsequently, paired-end sequencing was performed using the Illumina NovaSeq 6000 platform (Illumina Inc., San Diego, CA, USA). Through the sequencing process, 10 high concentrations and 30 low concentrations were generated, resulting in 40 paired-end RNA libraries. The raw and trimmed readings were inspected for quality using FastQC and MultiQC (Ewels et al. 2016), and Cutadapt was used to minimize adapter content and quality (Martin 2011).

### Sequencing data analysis

Preprocessing was performed by aligning the sequences using STAR (v.2.7.3a) (Dobin et al. 2013) and quantifying them using HTSeq (v.0.11.2) (Anders et al. 2015), where GRCh38 was used as the reference genome along with its annotation. While aligning the raw data (i.e., FASTQ files) through STAR, we optimally adjusted parameters such as “outFilterScoreMinOverLread” to 0 and “outFilterMatchNminOverLread” to 0.1 to maximize the number of uniquely mapped reads. Subsequently, we applied the transcripts per million method (Risso et al. 2014) with DGEobj.utils R package (Thompson et al. 2022) to the gene expression levels for normalization and carried out principal component analysis (PCA) (Karl Pearson 1901) with prcomp embedded in the stats R package and differential analyses including gene ontology (GO) analysis between the Low and High PM_2.5_ groups.

Differential expression analysis was conducted using the R package DESeq2 (Love et al. 2014). Significantly differentially expressed genes (DEGs) were visualized through volcano plots using the EnhancedVolcano R package (Kevin et al. 2021), and significantly enriched GO terms were shown as dot plots using the clusterProfiler R package (Wu et al. 2021). Two additional pathway enrichment analyses were performed: (i) Gene Set Enrichment Analysis (GSEA) (Subramanian et al. 2005; Mootha et al. 2003), whose visualization was achieved by mapping into Cytoscape (version 3.10.0) (Shannon et al. 2003) with the visualization app EnrichmentMap (Merico et al. 2010), and (ii) fast Gene Set Enrichment Analysis (FGSEA) (Korotkevich et al. 2021) illustrated through lollipop plots. For these analyses, two distinct gene sets were used, one of which was the whole aligned gene list and the other was the Mitocarta3.0 (Rath et al. 2021) gene list.

### Statistical analysis

All statistical analyses were performed using SPSS Software (version 21.0; IBM, Armonk, NY, USA). Descriptive statistics for the general characteristics of all participants (*n* = 454) recruited from the ongoing APPO study are presented in Table 1. Continuous variables are expressed as mean ± standard deviation, and categorical variables are expressed as total number (*n*) and percentage (%). To explore the association between PM_2.5_ levels and adverse pregnancy outcomes, multiple logistic regression was conducted, controlling for maternal age, pre-pregnancy BMI, education, income, birth weight, and infant sex.

**Table 1:** Characteristics of subjects (*n* = 454) selected from the APPO cohort between October 2020 and June 2022

Characteristics	Mean ± SD
Maternal age	33.6 ± 0.2
Pre-pregnancy BMI	22.3 ± 0.2
Gestational age at delivery	38.3 ± 0.1
Parity, *n* (%)	
First child	305 (67.2)
Second child	128 (28.2)
Third or following child	21 ( 4.6)
Adverse pregnancy outcomes, *n* (%)	
Preterm birth	44 ( 9.7)
Gestational diabetic milieu	41 ( 9.0)
Education level, *n* (%)	
≤ 12 years	41 ( 9.0)
> 12 years	412 (90.7)
No response	1 (0.2)
Income per month (USD)	
< 3050	101 (22.2)
3050 ~ 6099	123 (27.1)
> 6100	64 (14.1)
Unknown	44 ( 9.7)
No response	122 (26.9)
Smoking, *n* (%)	
Never smoker	413 (91.0)
Stopped smoking before pregnancy	40 ( 8.8)
Continued smoking during pregnancy	0
No response	1 ( 0.2)
In-house smoke exposure, *n* (%)	
No	431 (94.9)
Yes	23 ( 5.1)
Newborn sex, *n* (%)	
Male	247 (54.4)
Female	207 (45.6)
Birth weight	3139.4 ± 21.5
Ponderal index	2.6 ± 0.1
Apgar score 1 min	8.5 ± 0.1
Apgar score 5 min	9.4 ± 0.5
Exposure window of PM_2.5_, median (IQR)	
First trimester	8.8 (5.1)
Second trimester	9.5 (6.5)
Third trimester	11.3 (9.5)
Entire pregnancy	10.4 (7.5)

Data are shown as the mean ± SD for continuous variables and as *n* (%) for categorical data

*BMI* body mass index

## Results

### Environmental characteristics of the prospective APPO cohort study population

**Fig. 2:**
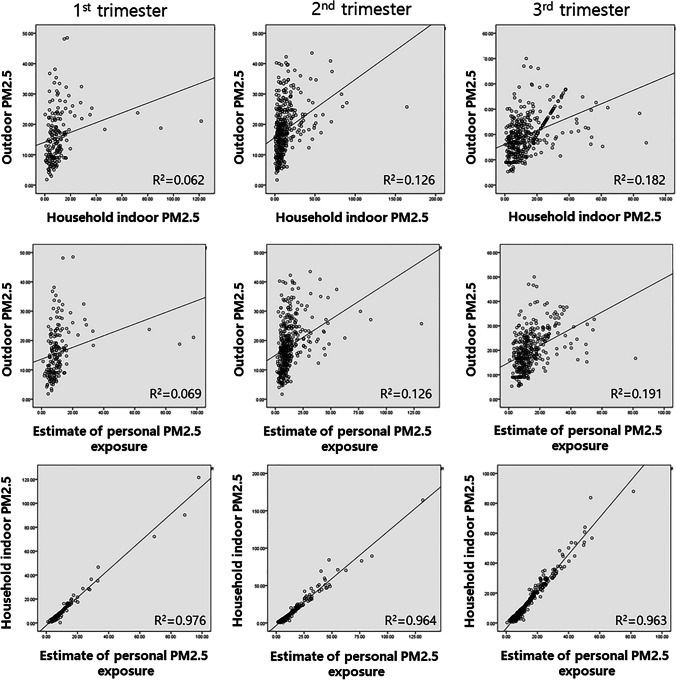
Correlation analysis between personal PM_2.5_ exposure, outdoor, and household indoor PM_2.5_ concentrations

We calculated the individual PM_2.5_ exposure of pregnant women using address-based outdoor PM_2.5_, household indoor PM_2.5_ concentrations, and time-activity analysis in the first, second, and third trimesters of pregnancy. Figure 2 shows the correlation between individual PM_2.5_ exposure and outdoor and household indoor PM_2.5_ concentrations in pregnant women. The positive correlations between address-based outdoor PM_2.5_ and indoor PM_2.5_ in every trimester were relatively low (first trimester, *r* = 0.062; second trimester, *r* = 0.126; third trimester, *r* = 0.182). However, individual PM_2.5_ exposure showed a significantly high positive correlation with household indoor PM_2.5_ concentrations (first trimester, *r* = 0.978; second trimester, *r* = 0.964; third trimester, *r* = 0.963). In the time-activity analysis, pregnant women spent more than 18 h at home, approximately 5 h indoors somewhere other than home, and approximately 1 h outdoors.

### Population characteristics of the prospective APPO cohort study

The general and lifestyle characteristics of the pregnant women are shown in Table 1. In total, 454 pregnant women were included with an average age of 33.6 years, a pre-pregnancy BMI of 22.3, and 67.2% of them were primiparous. Of the newborns, 54% were boys, with an average birth weight of 3139 g. Table 2 shows the results of the logistic regression of adverse pregnancy outcomes for every 5 µg/m^3^ increase in PM_2.5_ according to the exposure period during pregnancy. During the entire pregnancy or each trimester, PTB did not significantly increase after increases in the individual PM_2.5_. However, the incidence of gestational diabetes mellitus (GDM) significantly increased with every 5 µg/m^3^ increase PM_2.5_ in the third trimester and throughout pregnancy. After adjusting for maternal age, pre-pregnancy BMI, education, infant sex, and birth weight, PTB was significantly increased in the second trimester (odds ratio 1.391, *p* = 0.019), and GDM was significantly increased in the second (odds ratio 1.238, *p* = 0.041) and third (odds ratio 1.290, *p* = 0.029) trimesters, and entire PM_2.5_ level (odds ratio 1.295, *p* = 0.029).

**Table 2:** Logistic regression results of adverse pregnancy outcomes for every 5 µg/m^3^ increase in PM_2.5_

Exposure window	Unadjusted					Adjusted				
			95% CI					95% CI		
	*n*	Exp(B)	Lower	Upper	*p*-value	*n*	Exp(B)	Lower	Upper	*p*-value
Preterm birth										
First trimester	138	1.174	0.843	1.635	0.342	109	1.008	0.594	1.710	0.987
Second trimester	345	1.181	0.986	1.416	**0.071**	290	1.391	1.056	1.832	**0.019**
Third trimester	309	0.881	0.691	1.124	0.308	270	0.956	0.702	1.301	0.774
Entire	357	1.031	0.814	1.306	0.800	299	1.235	0.903	1.689	0.186
Gestational diabetes										
First trimester	138	0.739	0.360	1.515	0.409	109	0.538	0.167	1.740	0.301
Second trimester	345	1.193	0.995	1.431	**0.057**	290	1.238	1.008	1.519	**0.041**
Third trimester	309	1.264	1.030	1.552	**0.025**	270	1.290	1.026	1.622	**0.029**
Entire	357	1.258	1.026	1.543	**0.027**	299	1.295	1.027	1.632	**0.029**

Values in bold indicates significance at p <0.05

Adjusted factors: age, pre-pregnancy BMI, education, income, birth weight, infant sex

### Characteristics of subjects analyzed by mRNA sequencing in cord blood

To analyze the effects of PM_2.5_ on neonatal health through mRNA sequencing in cord blood, the characteristics of 40 pregnant women were randomly selected from the APPO study. The results are summarized in Table 3. There were no significant differences in maternal age, pre-pregnancy BMI, PTB, or GDM between the Low and High PM_2.5_ groups. However, PTB significantly increased in the second trimester (odds ratio 2.208, *p* = 0.027), following every 5 µg/m^3^ increase in the individual PM_2.5_ exposure (Supple. Table 1).

**Table 3:** Clinical and environmental characteristics of subjects (*n* = 40)

Characteristic	Low PM_2.5_	High PM_2.5_	*p-*value
	(*n* = 30)	(*n* = 10)	
Maternal age	32.9 ± 4.3	33.1 ± 4.9	0.920
Pre-pregnancy BMI	21.2 ± 2.6	21.8 ± 3.1	0.490
Gestational age at delivery	38.3 ± 1.9	37.4 ± 1.5	0.183
Parity, *n* (%)			0.337
First child	23 (76.7)	7 (70.0)	
Second child	6 (20.0)	2 (20.0)	
Third or following child	1 (3.3)	1 (10.0)	
Adverse pregnancy outcomes, *n* (%)			
Preterm birth	5 (16.7)	4 (40.0)	0.126
Gestational diabetic milieu	2 (6.7)	1 (10.0)	0.729
Smoking, *n* (%)			0.143
Never smoker	29 (96.7)	8 (80.0)	
Stopped smoking before pregnancy	1 (3.3)	2 (20.0)	
Continued smoking during pregnancy	0	0	
Newborn sex, *n* (%)			0.711
Male	13 (43.3)	3 (30.0)	
Female	17 (56.7)	7 (70.0)	
Birth weight	2999 ± 471.3	2844 ± 173.6	0.319
Ponderal index	2.6 ± 0.3	2.5 ± 0.3	0.134
Apgar score 1 min	8.5 ± 0.1	8.5 ± 0.1	0.200
Apgar score 5 min	9.4 ± 0.5	9.4 ± 0.5	0.943

Low PM_2.5_, pregnant women exposed to ≤ 15 µg/m^3^ of PM_2.5_ during pregnancy; High PM_2.5_, pregnant women exposed to > 15 µg/m^3^ of PM_2.5_ during pregnancy. Mann–Whitney *U* test for continuous variables (*p* < 0.05); *χ^2^ test (*p* < 0.05). Data are shown as the mean ± SD for continuous variables and as *n* (%) for categorical data

*BMI* body mass index

### DEGs between the High and Low PM_2.5_ groups

**Fig. 3:**
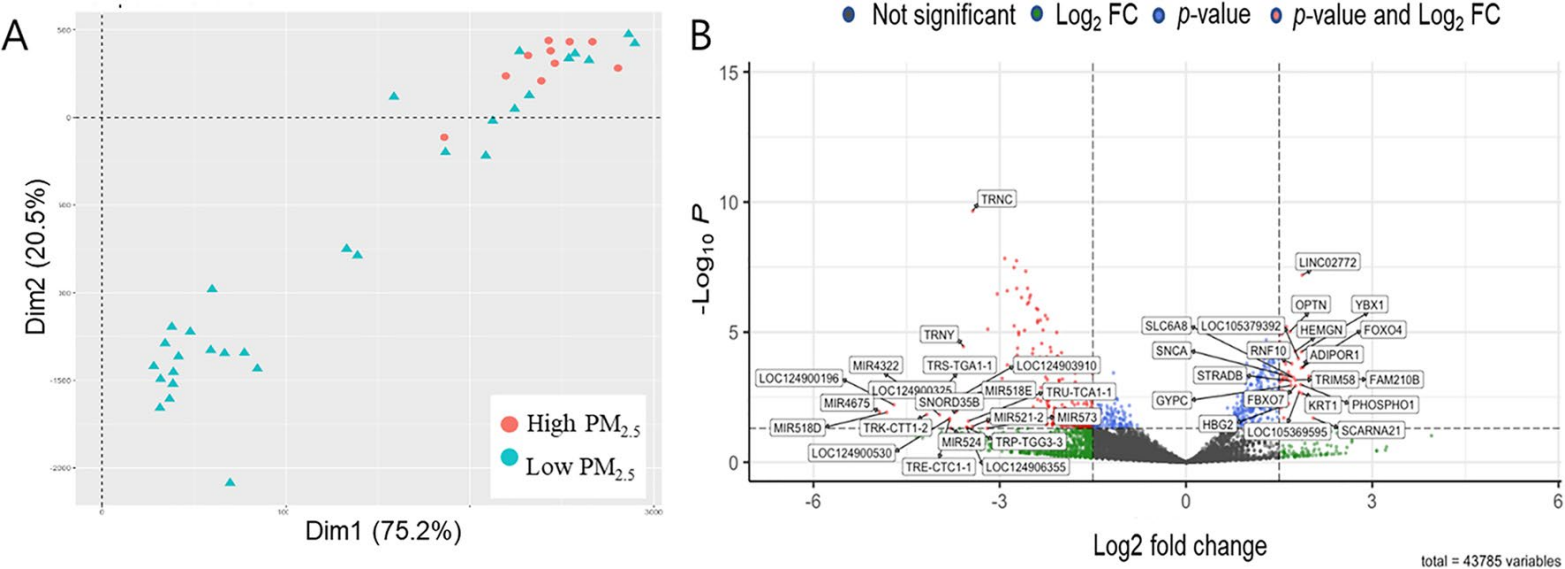
Principal component analysis plot and differentially expressed genes (DEGs) between the High PM_2.5_ (> 15 µg/m^3^ during pregnancy) and Low PM_2.5_ (≤ 15 µg/m^3^) groups. **A** PCA plot. **B** Volcano plot. Dotted lines represent the cut-offs for the log2 fold change (1.5) and *p*-value (0.05). Red and green dots denote the 48 upregulated and 322 downregulated genes in the High PM_2.5_ group, respectively

Principal component analysis of the RNA-seq data showed that the High PM_2.5_ group (*n* = 10) was generally distinguished from the Low PM_2.5_ group (*n* = 30) (Fig. 3A). Through the analysis of DEGs between the two groups, we first collected 4375 genes expressed in either the High or Low PM_2.5_ group, and identified 370 DEGs (48 upregulated and 322 downregulated genes) in the High PM_2.5_ group (i.e., *p*-value < 0.05, and a log2 fold change > 1.5, Supple. Table 2), as shown in Fig. 3B. Among the top genes that were significantly upregulated in the High PM_2.5_ group, some genes, such as FAM210B, KRT1, FOXO4, TRIM58, and FBXO7 were found to be involved in mitochondria-associated activity, whereas others such as ADIPOR1, OPTN, HBG2, YBX1 (YB-1), and NFkB1 were associated with common obstetric issues, including PTB. The top genes significantly downregulated in the High PM_2.5_ group, including PF4V1, PF4, and S100A9 were found to be associated with inflammatory processes (Fig. 3B).

### Enriched pathways in the High and Low PM_2.5_ groups

**Fig. 4:**
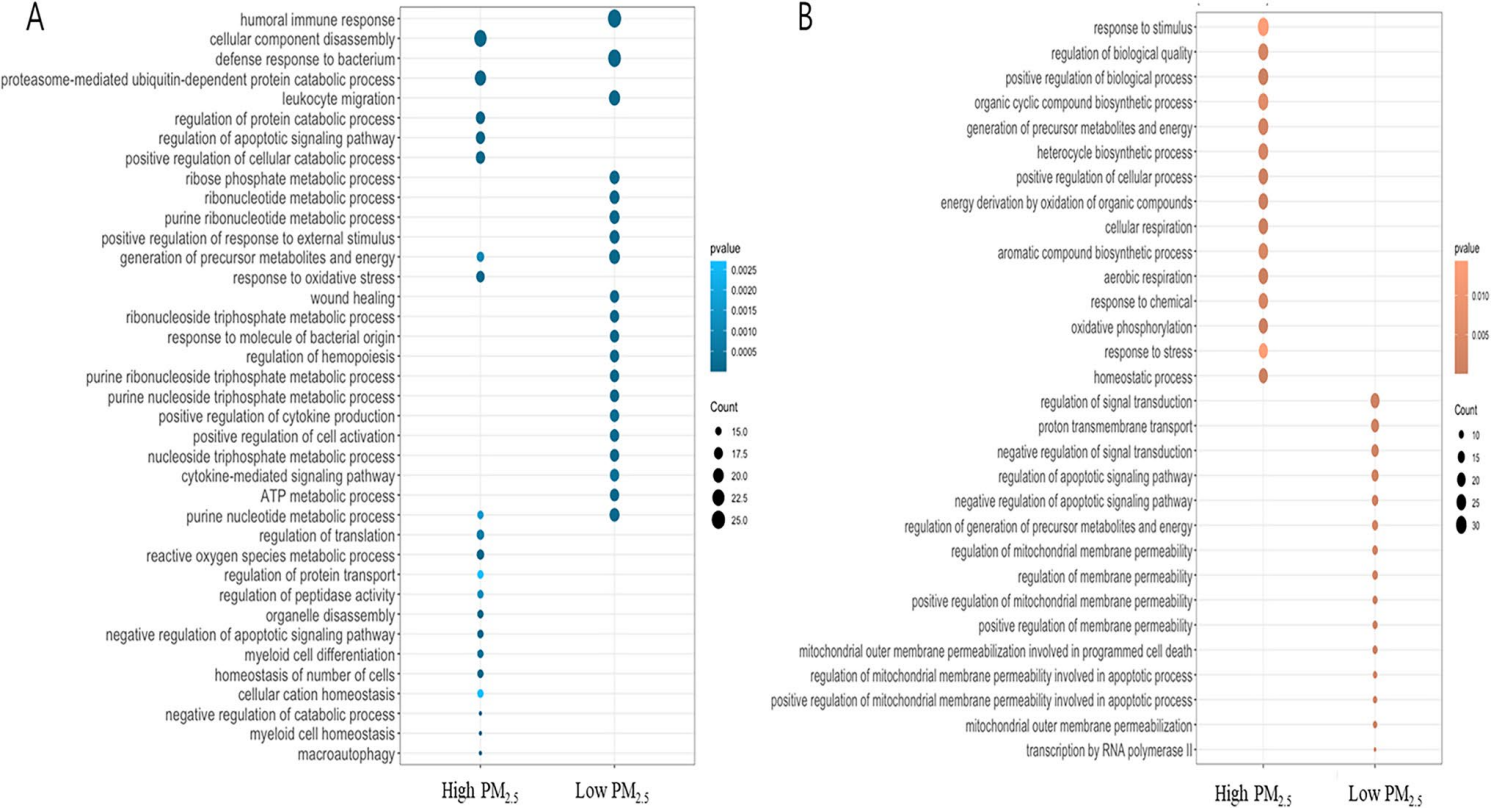
Enriched pathways in the High and Low PM_2.5_ groups. Gene ontology biological processes (GOBPs) enriched by the genes that were up- and downregulated in the High PM_2.5_ compared with that in the Low PM_2.5_ group. A Dot plot of the enriched pathways in the two groups. The significant genes for the enrichment analysis were selected with *p*-value < 0.05, and the absolute value of log2 fold-change > 0.2 for all genes in the pathways were selected with *p*-value < 0.05 and *q*-value < 0.1. B The significant genes within the MitoCarta 3.0 gene list were selected with *p*-value < 0.5, based on the results of differential expression analysis. The pathways were selected with a *p*-value < 0.05 and *q*-value < 0.1. The blue color density is represented by the significance (*p*-value) of the GOBPs, and the circle size revealed the effect size

**Fig. 5:**
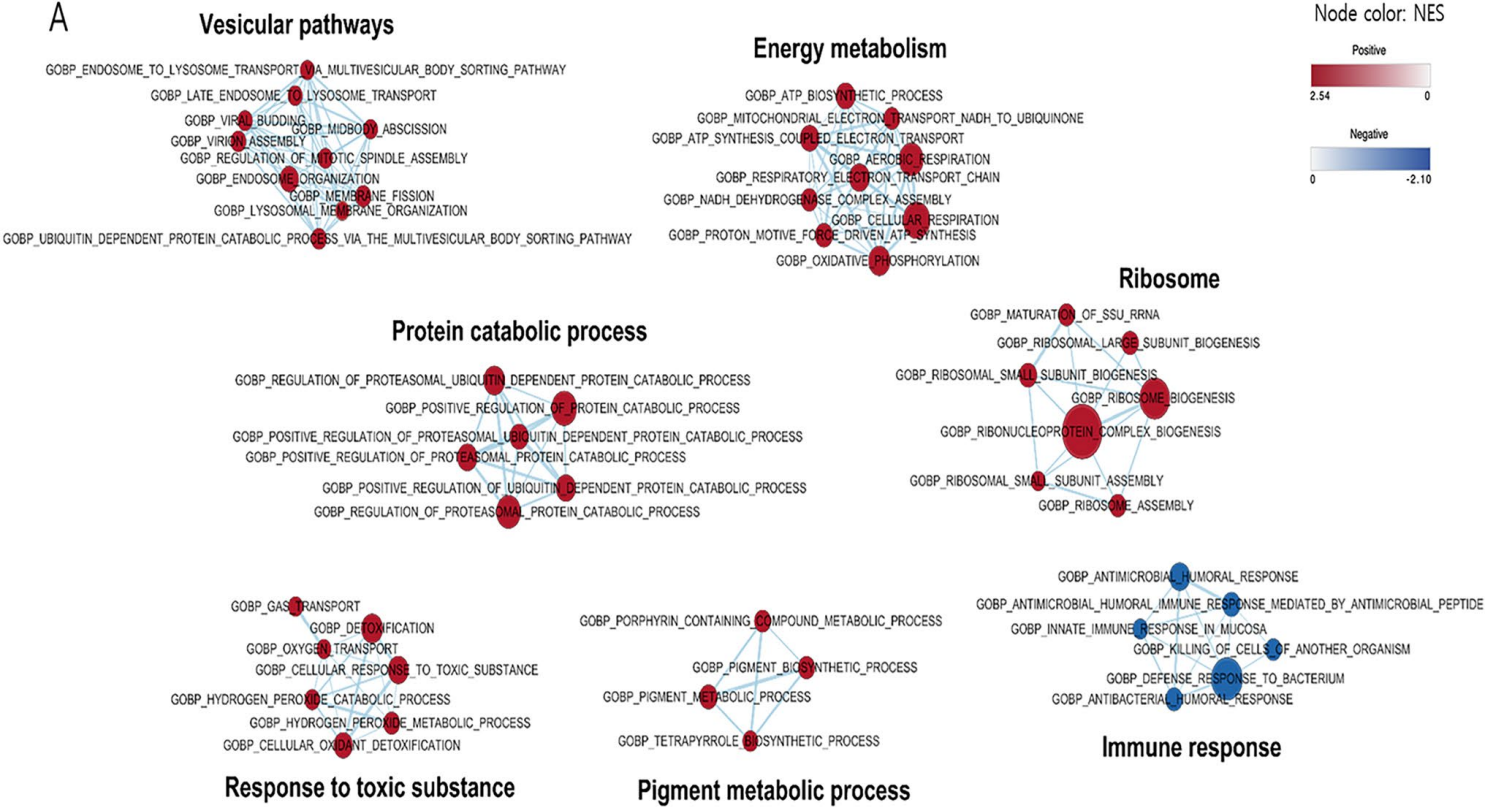

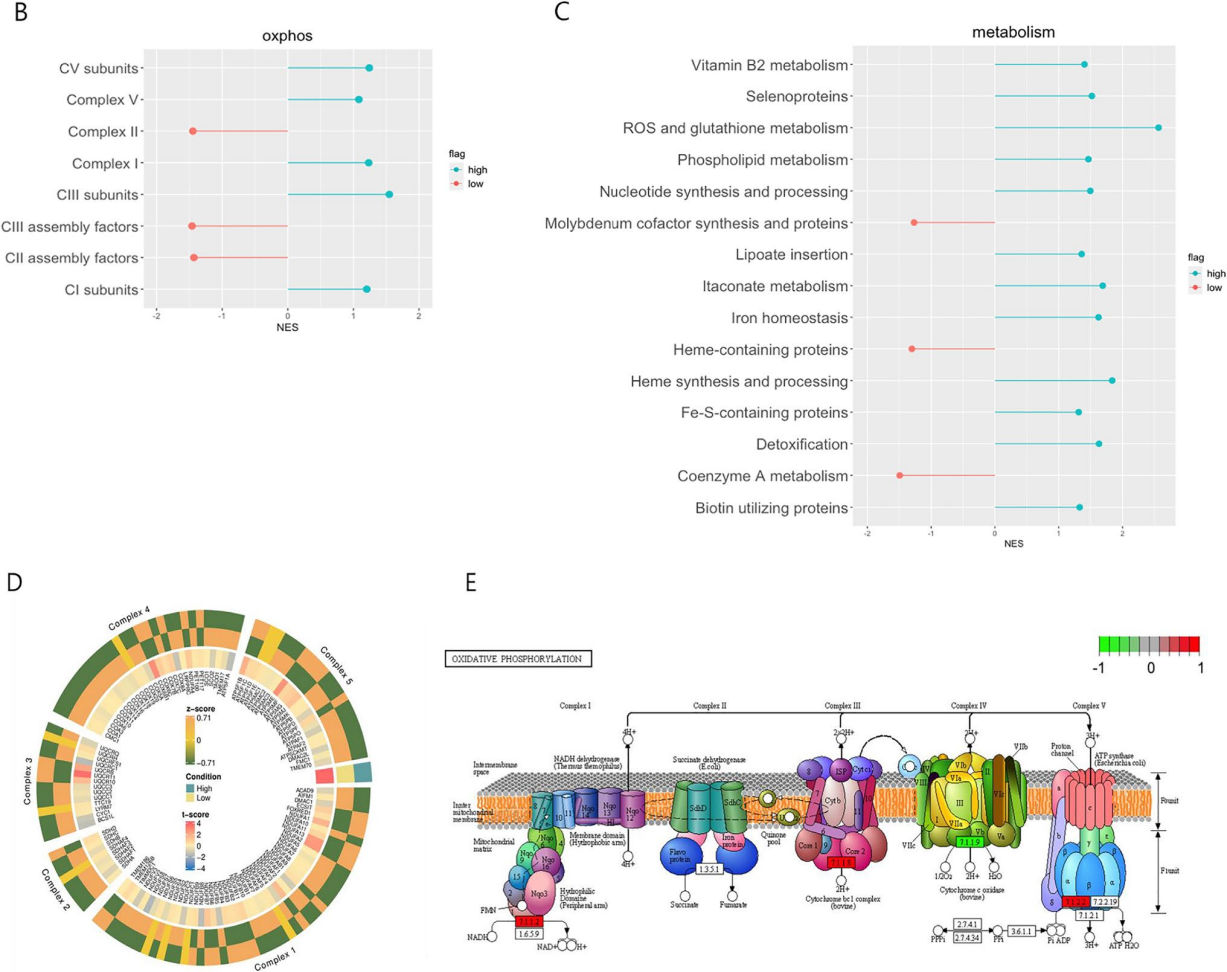
Network of enriched pathways and lollipop plots involving mitochondria in the High and Low PM_2.5_ groups. **A** Network figure of the enriched pathways in the two groups. The red nodes indicate upregulated pathways in the High PM_2.5_ group and the blue nodes denote upregulation in the Low PM_2.5_ group. The selected pathways were filtered for FDR < 0.1 and edge similarity > 0.375. **B** OXPHOS pathways in High and Low PM_2.5_ groups. **C** Mitochondrial metabolic pathways in the High and Low PM_2.5_ groups, which exhibited the top 15 absolute NES values. **D** Heatmap of the *z*- and *t*-scores of genes associated with OXPHOS in the High and Low PM_2.5_ groups. **E** Path view of OXPHOS pathway with MitoCarta genes (*p*-value < 0.5) based on KEGG pathways. The red box indicates upregulation and green box indicates downregulation

We examined cellular processes by GSEA-based network analysis, illustrated in Fig. 4A, which displayed seven clusters, where six of them were in the High PM_2.5_ group and only one was in the Low PM_2.5_ group. The six clusters were composed of vesicular pathways, protein catabolic process, response to toxic substance, pigment metabolic process, energy metabolism, and ribosome. While the upregulated clusters of the High PM_2.5_ group represented consistent findings including mitochondria-associated process (i.e., cellular respiration and OXPHOS) and protein catabolic process. The only upregulated cluster of the Low PM_2.5_ group, however, showed pathways associated with immune response which was in line with the previously described GO analysis. FGSEA also demonstrated that OXPHOS and other direct/indirect associated pathways were enhanced in the High PM_2.5_ group. As shown in Fig. 4B, complex I, complex I subunits, complex V, complex V subunits, and complex III subunits were upregulated in the High PM_2.5_ group among the protein complexes that are associated with OXPHOS. In the metabolism-associated pathways, ROS and glutathione metabolism, nucleotide synthesis and processing, and iron homeostasis were upregulated in the High PM_2.5_ group, and heme-containing proteins, and coenzyme A metabolism were upregulated in the Low PM_2.5_ group (Fig. 5C). Interestingly, PTBs (*n* = 3) in the High PM_2.5_ group were upregulated in OXPHOS‑associated pathways compared with the full term delivered group with the 10 lowest concentrations (Supple. Figure 1).

## Discussion

The main finding of this study was that exposure to high concentrations of fine particulate matter during pregnancy was associated with an increased risk of PTB by upregulating PTB-related genes and mitochondrial OXPHOS dysfunction due to complex subunits alterations. This is the first prospective pregnancy cohort study to identify the mechanism of the association among PM_2.5_ exposure, PTB, and neonatal health threats. Furthermore, our findings suggest that exposure to high levels of PM_2.5_ during pregnancy may congenitally alter mitochondrial OXPHOS in preterm infants as well as in full term newborns.

Fine particulate matter, major ambient air pollutant, is a complex mixture of organic and inorganic particles. Maternal exposure to ambient PM_2.5_ can increase the risk of adverse pregnancy outcomes, including PTB, GDM, low birth weight, and small for gestational age. People typically spend up to 90% of their day indoors (Selevan et al. 2000; Zhu et al. 2015); therefore, indoor PM concentrations have an important impact on individual exposure. It has been recorded that indoor PM concentrations can exceed outdoor concentrations (Klepeis et al. 2001). The sources of indoor PM include aerosol sprays, cooking, candle burning, heating/cooking with solid fuels, improperly tuned gas stoves and furnaces, pets, dust mites, cleaning, and smoking. Indoor air is also affected by the infiltration of outdoor air (Goyal and Kumar 2013), potentially affecting PM concentrations. Depending on the outdoor PM levels, this infiltration can cause an increase or decrease in indoor PM levels (Hegde et al. 2020).

This study used a time-weighted average model with household indoor and outdoor PM_2.5_ concentrations, and time-activity records to calculate individual PM_2.5_ exposure and found that every 5 µg/m^3^ increase was associated with a higher risk of PTB and GDM. The individual PM_2.5_ exposure was most affected by household indoor PM_2.5_ concentrations because pregnant women spent more than 95% of their time indoors. However, individual exposure estimates were not affected by home-address-based outdoor PM_2.5_. None of the pregnant women smoked during pregnancy, and only 5% were exposed to household smoking, suggesting that the contribution was minimal. Future studies should investigate the sources of indoor PM_2.5_ and analyze its association with pregnancy outcomes.

Adverse pregnancy outcomes due to maternal exposure to fine PM affect fetal growth and development (Zhu et al. 2015). Low birth weight and PTB are well known to be associated with increased neonatal morbidity and mortality as well as possible increased morbidity in adulthood (Behrman and Butler 2007). These adverse pregnancy outcomes from PM_2.5_ exposure are primarily related to their ability to induce oxidative stress and inflammation (Jiang et al. 2019; Kamdar et al. 2008; Møller et al. 2014). The large surface area of PM_2.5_ and the presence of heavy metals adsorbed on its surface can generate higher amounts of hydroxyl radicals compared to larger particles (PM_10_) (Kumar and Morawska 2013; Morawska et al. 2001). Studies have shown that environmental pollutants primarily target mitochondria and have several detrimental effects. Several studies have linked in-utero PM exposure to mitochondrial oxidative dysfunction (Grevendonk et al. 2016; Brunst et al. 2018; Cosemans et al. 2022). One study reported that PM_10_ exposure increased mitochondrial 8-hydroxy-2′-deoxyguanosine (8-OHdG) levels in maternal and cord blood, which was associated with increased systemic oxidative stress at the mitochondrial level (Grevendonk et al. 2016). Another study reported that increased exposure to PM_2.5_ during pregnancy was associated with decreased mitochondrial DNA copy number (mtDNAcn) in the cord blood, depending on the fetal sex (Brunst et al. 2018). Another study reported that in-utero exposure to PM_2.5_ during the first trimester of pregnancy was associated with cord blood MT-ND4L_10550A>G_ heteroplasmy in newborns, which was associated with higher childhood weight (Cosemans et al. 2022). Although many studies have been conducted on mitochondrial dysfunction and fine particles, there is a significant lack of studies reporting the mechanisms between maternal exposure to PM_2.5_, and redox imbalance mechanisms in neonatal cord blood.

The results of mRNA-seq analysis in cord blood showed that among the top gene list with significant log2 fold changes, only FAM210B, a mitochondrial protein, is known to be linked to erythroid differentiation (Kondo et al. 2016), whereas KRT1 and FOXO4 are associated with oxidative stress (Yang et al. 2022; Collard et al. 2001). Also, TRIM58 and FBXO7 are involved in the ubiquitin-dependent protein catabolic pathway (Lee et al. 2023). Using DEG, three pathway enrichment analyses, including GO, GSEA, and FGSEA, revealed that the enriched pathways in the High PM_2.5_ group were mainly involved in mitochondrial- and apoptosis-related pathways. In addition, changes in mitochondrial activity and ribosome assembly suggest a possible alteration in proteostasis by maintaining proteome homeostasis (Lu and Guo 2020). In contrast, pathways related to the immune response and inflammatory mechanisms were enriched in the Low PM_2.5_ group. Further, GO analysis using Mitokarta 3.0 showed upregulation of energy metabolism, mainly through precursor metabolites and energy production, energy derivation by oxidation of organic compounds, cellular respiration, aerobic respiration, and OXPHOS.

With the development of next-generation sequencing techniques, the number of genes with mutations known to cause mitochondrial diseases has increased substantially (Carroll et al. 2014). To date, mutations have been described in nuclear genes encoding OXPHOS structural proteins, as well as factors involved in virtually every step of OXPHOS biogenesis, including mtDNA replication and maintenance, mitochondrial transcription and translation, import, assembly, synthesis, and incorporation of redox cofactors, as well as proteins required for proper mitochondrial cristae shape, lipid milieu composition, and detoxification pathways (Fernandez-Vizarra and Zeviani 2021). In OXPHOS disorders caused by mutations in the structural subunits and assembly factors, the severity of biochemical and assembly defects is highly variable and largely depends on the location of the protein in the assembly process and the nature of the mutation (Fernandez-Vizarra et al. 2009; Ghezzi and Zeviani 2018).

Mitochondrial DNA encodes the proteins of the electron transport chain (ETC; 13 subunits of complexes I, III, and IV; and ATP synthase (complex V)) that are essential for OXPHOS (Taanman 1999). During mitochondrial respiration, electrons from complexes I and III react with molecular oxygen to form superoxides, which damage ROS (Andreyev et al. 2005). Because of its lack of histones, which protect against oxidative stress, and its close proximity to the ETC, the primary source of ROS, mtDNA is more vulnerable to the accumulation of ROS-induced damage than nuclear DNA (Ballinger et al. 2000; Tatarenkov and Avise 2007). In our results, the High PM_2.5_ group had upregulated complex I, complex I subunits, complex V, complex V subunits, and complex III subunits. These findings suggest that exposure to high levels of fine particulate matter may affect the ETC complex and structural subunits.

The Low PM_2.5_ group showed upregulated complex II and coenzyme A metabolism. Complex II (succinic dehydrogenase, SDH) is an enzyme involved in the ETC and the Krebs cycle that oxidizes succinate to fumarate and transfers electrons to CoQ. The pathogenesis of mutations and deficiencies in the CII subunit appears to be related to succinate accumulation, which is associated with a control mechanism that activates the hypoxic program of cells (Selak et al. 2005). Complex III constitutes the central part of the ETC, which accepts two electrons from reduced CoQ (CoQH2) and donates them to cytochrome c. Mutations and deficiencies in the CIII subunit are associated with sporadic myopathy, exercise intolerance, recurrent metabolic crisis, insulin-responsive hyperglycemia, and lactic acidosis (Gaignard et al. 2013; Gusic et al. 2020). Thus, our results suggest that despite limitations in the literature, high levels of PM_2.5_ in mother-neonate pairs may affect mitochondrial oxidative stress dysfunction in newborns, which may be involved in their growth and later development of disease.

While our transcriptomic analysis primarily revealed mitochondrial-associated changes, other important alterations were also demonstrated and significant changes were identified, with some genes associated with obstetrical issues, consistent with previous studies. In fetal and placental development, YB-1 has been shown to play a crucial role during the gestation period (Meyer et al. 2020). For uterine or pregnancy-related functions, ADIPOR1 has been proposed to influence uterine contractility, suggesting a possible connection between plasma membrane adiponectin receptors and preterm birth (Vyas et al. 2019). Also, OPTN has been associated with nuclear factor-kappa B activity (Akizuki et al. 2013), and NFkB1 and HBG2 have been directly/indirectly involved in PTB (Pique-Regi et al. 2019; Fang et al. 2022). Additionally, while inflammatory-associated pathways in the Low PM_2.5_ group and cell-death-associated pathways in the High PM_2.5_ group were both upregulated, ribonucleoside triphosphate metabolic process in the Low PM_2.5_ group and proteasome-mediated ubiquitin-dependent protein catabolic process in the High PM_2.5_ group are known to be associated with Nrf2 signaling pathways (Gao et al. 2020). Therefore, we propose that exposure to high levels of fine particulate matter during pregnancy can increase the risk of PTB by affecting uterine contractions, NFkB activity, and cell death. We further propose that alterations in mitochondrial OXPHOS caused by exposure to high levels of PM have health consequences for both preterm and neonatal infants.

The limitations of this study are that although most pregnant women spent a lot of time indoors at home, the sample size was small and household indoor PM_2.5_ could not be measured during the entire pregnancy period. In addition, the identification of PM_2.5_-related genes in cord blood requires validation in a larger population. Nevertheless, the strength of this study is reported the association between PM_2.5_, PTB, and GDM in a maternal health effect analysis using personal PM_2.5_ exposure through IOT-based indoor and addressee-based outdoor PM_2.5_ measurement, and time-activity pattern.

## Conclusion

In an ongoing prospective cohort, the APPO study, PTB and GDM were associated with every 5 µg/m^3^ increase in individual PM_2.5_ exposure. In addition, neonatal cord blood samples from high PM_2.5_ exposure may induce dysfunction of genes associated with common obstetric problems, including PTB, and mitochondrial OXPHOS dysfunction through changes in some ETC complex proteins. This suggests that maternal exposure to fine particulate matter affects PTB-related gene activity, growth, and subsequent health via mitochondrial impairment in preterm infants, including newborns. Further analysis of the sources of household indoor PM and composition of household indoor PM in relation to oxidative damage is needed to support this study, and further studies with larger cohorts are warranted.

### Supplementary Information

Below is the link to the electronic supplementary material.

Supplementary file1 (DOCX 352 KB)

## Data Availability

All data generated or analyzed during this study are included in this published article [and its additional information files]. Raw data of mRNA sequencing will be made available on request.

## Abbreviations

PM2.5: Fine particulate matter
PTB: Preterm birth
GDM: Gestational diabetes mellitus
OXPHOS: Oxidative phosphorylation
ROS: Reactive oxygen species
BMI: Body mass index
hs-CRP: High-sensitivity C-reactive protein
PCA: Principal component analysis
DEG: Differentially expressed gene
FGSEA: Fast gene set enrichment analysis
mtDNA: Mitochondrial DNA
ETC: Electron transport chain
