# Supplementary material for: Maternal PM2.5 exposure is associated with preterm birth and gestational diabetes mellitus, and mitochondrial OXPHOS dysfunction in cord blood
Source: Environ Sci Pollut Res Int. 2024 Jan 10;31(7):10565–78. doi: 10.1007/s11356-023-31774-0 (PMC10850187; doi:10.1007/s11356-023-31774-0)
Supplement: Supplementary file 1 — Supplementary file1 (DOCX 352 KB) [file 11356_2023_31774_MOESM1_ESM.docx]

**Supplementary data**

**Maternal PM2.5 Exposure is associated with Preterm Birth and Gestational Diabetes Mellitus, and Mitochondrial OXPHOS Dysfunction in Cord Blood**

Young-Ah You ^1†^, Sunwha Park^1^, Eunjin Kwon^2^, Ye-Ah Kim ^3,4^, Young Min Hur^1^, Ga In Lee^1^, Soo Min Kim^1^, Jeong Min Song ^3,5^, Man S Kim^3**^, Young Ju Kim^1*^, The APPO cohort study group^#^

^1^ Department of Obstetrics and Gynecology, College of Medicine and Ewha Medical Institute, Ewha Womans University, Seoul 07804, Republic of Korea

^2^ Division of Allergy and Respiratory Disease Research, Department of chronic disease convergence, National Institute of Health, Cheongju 28159, Republic of Korea

^3^ Translational-Transdisciplinary Research Center, Clinical Research Institute, Kyung Hee University Hospital at Gangdong, College of Medicine, Kyung Hee University, Seoul, Republic of Korea

^4^ Department of Biomedical Science and Technology, Graduate School, Kyung Hee University, Seoul, Republic of Korea

^5^ Department of Obstetrics and Gynecology, Kyung Hee University at Gangdong, Seoul, Republic of Korea


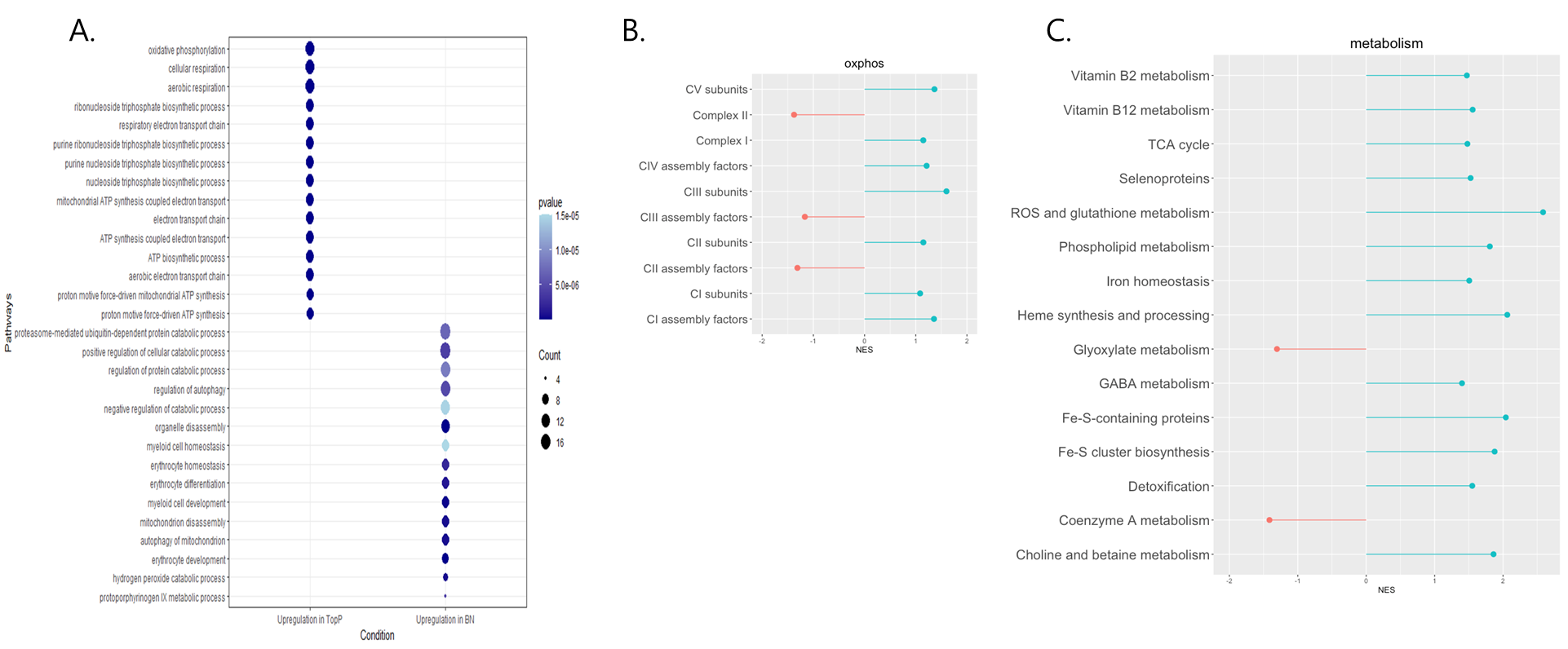


Supplementary Figure 1. Enriched pathways and lollipop plots involving mitochondria in preterm birth samples of the High PM_2.5_ (> 15 ug/m^3^ during pregnancy) and Low PM_2.5_ (≤ 15 ug/m^3^) groups. **A.** Enriched pathways in the two groups using MitoCarta3.0 analysis. **B**. Oxidative phosphorylation pathways. **C**. Mitochondrial metabolic pathway

Supplementary Table 1. Logistic regression results of adverse pregnancy outcomes for every 5 ug/m^3^ increase in PM_2.5_

|  | Unadjusted | |  |  |  | Adjusted |  |  |  |  |
| --- | --- | --- | --- | --- | --- | --- | --- | --- | --- | --- |
|  |  |  | 95% CI | |  |  |  | 95% CI | |  |
|  | n | Exp(B) | Lower | Upper | p-value | n | Exp(B) | Lower | Upper | p-value |
| PTB |  |  |  |  |  |  |  |  |  |  |
| Second trimester | 32 | 2.208 | 1.093 | 4.461 | **0.027** | 32 | 2.731 | 1.166 | 6.396 | **0.021** |
| Third trimester | 30 | 1.005 | 0.602 | 1.675 | 0.986 | 30 | 0.983 | 0.573 | 1.685 | 0.950 |
| Entire | 32 | 1.529 | 0.740 | 3.16 | 0.252 | 32 | 1.377 | 0.639 | 2.964 | 0.414 |
| GDM |  |  |  |  |  |  |  |  |  |  |
| Second trimester | 32 | 1.611 | 0.753 | 3.448 | 0.219 | 32 | 1.845 | 0.71 | 4.793 | 0.209 |
| Third trimester | 30 | 0.985 | 0.462 | 2.103 | 0.969 | 30 | 0.975 | 0.445 | 2.136 | 0.950 |
| Entire | 32 | 3.273 | 0.886 | 12.088 | **0.075** | 32 | 2.977 | 0.724 | 12.247 | 0.131 |
| Adjusted factors: Age, Pre-pregnancy BMI, Education, Income, Birth weight, Infant sex | | | | | | | |  |  |  |

Supplementary Table 2. Differential expressed genes between High PM_2.5_ and Low PM_2.5_

| No | Gene symbol | Average of normalized expression | | log2-Fold Change | p-value |
| --- | --- | --- | --- | --- | --- |
|  |  | HC | LC |  |  |
| 1 | SCARNA21 | 55 | 40 | 2.05 | 0.0202 |
| 2 | FAM210B | 1888 | 1446 | 1.98 | 0.0005 |
| 3 | ADIPOR1 | 1670 | 1371 | 1.89 | 0.0002 |
| 4 | LINC02772 | 82 | 71 | 1.87 | 0.0000 |
| 5 | KRT1 | 1563 | 1296 | 1.87 | 0.0023 |
| 6 | YBX1 | 459 | 383 | 1.86 | 0.0001 |
| 7 | FOXO4 | 405 | 342 | 1.85 | 0.0002 |
| 8 | PHOSPHO1 | 1801 | 1516 | 1.85 | 0.0010 |
| 9 | LOC105369595 | 202 | 172 | 1.82 | 0.0020 |
| 10 | LOC105379392 | 146 | 126 | 1.81 | 0.0001 |
| 11 | FBXO7 | 785 | 697 | 1.76 | 0.0012 |
| 12 | TRIM58 | 463 | 413 | 1.76 | 0.0007 |
| 13 | HEMGN | 241 | 220 | 1.76 | 0.0001 |
| 14 | SNCA | 245 | 224 | 1.73 | 0.0005 |
| 15 | RNF10 | 267 | 252 | 1.70 | 0.0002 |
| 16 | GYPC | 552 | 519 | 1.69 | 0.0011 |
| 17 | STRADB | 308 | 292 | 1.68 | 0.0008 |
| 18 | SLC6A8 | 314 | 299 | 1.68 | 0.0005 |
| 19 | OPTN | 110 | 114 | 1.67 | 0.0000 |
| 20 | HBG2 | 2315109 | 2219504 | 1.65 | 0.0057 |
| 21 | DMTN | 392 | 380 | 1.65 | 0.0003 |
| 22 | YBX3P1 | 505 | 497 | 1.63 | 0.0006 |
| 23 | CCNI | 180 | 180 | 1.62 | 0.0000 |
| 24 | FURIN | 210 | 211 | 1.60 | 0.0002 |
| 25 | UBBP4 | 102 | 102 | 1.60 | 0.0006 |
| 26 | LOC105378469 | 9 | 8 | 1.58 | 0.0199 |
| 27 | SMIM1 | 196 | 198 | 1.57 | 0.0019 |
| 28 | FAM104A | 108 | 113 | 1.57 | 0.0001 |
| 29 | RUNDC3A | 130 | 132 | 1.57 | 0.0020 |
| 30 | ALAS2 | 2055 | 2088 | 1.57 | 0.0038 |
| 31 | YOD1 | 170 | 175 | 1.55 | 0.0006 |
| 32 | EIF1B | 729 | 749 | 1.55 | 0.0007 |
| 33 | SLC14A1 | 61 | 65 | 1.55 | 0.0000 |
| 34 | ATP6V0C | 2207 | 2306 | 1.54 | 0.0017 |
| 35 | MBNL3 | 46 | 50 | 1.54 | 0.0000 |
| 36 | FKBP8 | 5795 | 6053 | 1.54 | 0.0064 |
| 37 | DCAF12 | 331 | 347 | 1.53 | 0.0039 |
| 38 | GABARAPL2 | 296 | 311 | 1.53 | 0.0004 |
| 39 | OR2W3 | 320 | 333 | 1.53 | 0.0081 |
| 40 | MXI1 | 82 | 88 | 1.53 | 0.0001 |
| 41 | TENT5C | 712 | 747 | 1.53 | 0.0035 |
| 42 | ASCC2 | 79 | 88 | 1.53 | 0.0000 |
| 43 | SRXN1 | 61 | 65 | 1.53 | 0.0001 |
| 44 | YBX3 | 636 | 674 | 1.52 | 0.0030 |
| 45 | WDR26 | 65 | 71 | 1.51 | 0.0000 |
| 46 | WASF2 | 43 | 48 | 1.51 | 0.0000 |
| 47 | GSPT1 | 134 | 146 | 1.51 | 0.0008 |
| 48 | CNPPD1 | 391 | 414 | 1.51 | 0.0039 |
| 49 | TMEM125 | 2 | 25 | -1.50 | 0.0420 |
| 50 | LINC02908 | 3 | 31 | -1.50 | 0.0373 |
| 51 | JUNB | 115 | 988 | -1.51 | 0.0004 |
| 52 | MYD88 | 9 | 225 | -1.51 | 0.0060 |
| 53 | FOS | 48 | 414 | -1.51 | 0.0020 |
| 54 | CFP | 9 | 105 | -1.51 | 0.0154 |
| 55 | PIF1 | 4 | 40 | -1.52 | 0.0371 |
| 56 | LOC124902993 | 3 | 34 | -1.52 | 0.0469 |
| 57 | PYCARD | 9 | 79 | -1.53 | 0.0134 |
| 58 | TIMP1 | 16 | 141 | -1.53 | 0.0047 |
| 59 | LOC124904744 | 2 | 27 | -1.53 | 0.0484 |
| 60 | TGFB1 | 11 | 97 | -1.53 | 0.0075 |
| 61 | CRYBB3 | 1 | 21 | -1.53 | 0.0472 |
| 62 | LOC124909476 | 3 | 31 | -1.53 | 0.0361 |
| 63 | CHAD | 4 | 43 | -1.53 | 0.0482 |
| 64 | LOC107987269 | 1 | 21 | -1.53 | 0.0448 |
| 65 | P2RY13 | 9 | 99 | -1.53 | 0.0181 |
| 66 | LOC105371253 | 9 | 323 | -1.54 | 0.0105 |
| 67 | LOC105369743 | 1 | 21 | -1.55 | 0.0414 |
| 68 | ARHGDIB | 19 | 168 | -1.55 | 0.0015 |
| 69 | BCL6B | 4 | 44 | -1.55 | 0.0420 |
| 70 | CD14 | 12 | 115 | -1.55 | 0.0033 |
| 71 | LINC00452 | 2 | 27 | -1.55 | 0.0399 |
| 72 | TCL1B | 2 | 27 | -1.55 | 0.0399 |
| 73 | DYNLT4 | 2 | 25 | -1.56 | 0.0481 |
| 74 | LOC124903470 | 2 | 25 | -1.56 | 0.0499 |
| 75 | GPRC5D | 1 | 73 | -1.56 | 0.0394 |
| 76 | LOC124901906 | 1 | 22 | -1.56 | 0.0452 |
| 77 | LINC01741 | 1 | 22 | -1.56 | 0.0452 |
| 78 | HDGFL1 | 1 | 111 | -1.56 | 0.0408 |
| 79 | TCF15 | 1 | 60 | -1.56 | 0.0426 |
| 80 | TRNT | 45 | 399 | -1.56 | 0.0184 |
| 81 | LOC124900937 | 1 | 173 | -1.57 | 0.0470 |
| 82 | LOC107984302 | 11 | 916 | -1.57 | 0.0119 |
| 83 | LOC124902719 | 2 | 26 | -1.57 | 0.0320 |
| 84 | TBC1D27P | 1 | 21 | -1.58 | 0.0387 |
| 85 | CCDC183-AS1 | 1 | 21 | -1.58 | 0.0387 |
| 86 | SPATA31C2 | 2 | 27 | -1.58 | 0.0341 |
| 87 | LRRC37A6P | 1 | 21 | -1.58 | 0.0402 |
| 88 | RAMP3 | 2 | 26 | -1.58 | 0.0305 |
| 89 | LOC107984635 | 1 | 21 | -1.58 | 0.0436 |
| 90 | LOC105371155 | 1 | 21 | -1.59 | 0.0436 |
| 91 | LOC124905090 | 1 | 21 | -1.59 | 0.0450 |
| 92 | TLE7 | 2 | 28 | -1.59 | 0.0375 |
| 93 | OTUD6A | 3 | 125 | -1.59 | 0.0374 |
| 94 | FPR1 | 9 | 83 | -1.59 | 0.0064 |
| 95 | B2M | 20 | 182 | -1.59 | 0.0028 |
| 96 | LOC100996549 | 2 | 27 | -1.59 | 0.0303 |
| 97 | TYROBP | 18 | 168 | -1.60 | 0.0015 |
| 98 | RGS18 | 10 | 92 | -1.60 | 0.0125 |
| 99 | FRAT2 | 9 | 86 | -1.60 | 0.0050 |
| 100 | GPR85 | 68 | 1025 | -1.60 | 0.0017 |
| 101 | SRGN | 13 | 120 | -1.60 | 0.0028 |
| 102 | IGKC | 52 | 476 | -1.61 | 0.0330 |
| 103 | PLPP2 | 3 | 38 | -1.61 | 0.0416 |
| 104 | PHOX2A | 3 | 39 | -1.61 | 0.0471 |
| 105 | LOC105369209 | 1 | 22 | -1.61 | 0.0317 |
| 106 | L3MBTL4-AS1 | 2 | 27 | -1.61 | 0.0287 |
| 107 | LOC124900983 | 2 | 29 | -1.62 | 0.0356 |
| 108 | LOC124903508 | 4 | 48 | -1.62 | 0.0387 |
| 109 | CFL1 | 55 | 511 | -1.63 | 0.0002 |
| 110 | TBATA | 1 | 22 | -1.63 | 0.0303 |
| 111 | LINC02918 | 1 | 58 | -1.63 | 0.0312 |
| 112 | CRB3 | 1 | 99 | -1.63 | 0.0355 |
| 113 | LOC124903670 | 3 | 38 | -1.63 | 0.0367 |
| 114 | LOC124902369 | 2 | 29 | -1.63 | 0.0410 |
| 115 | LOC107986031 | 2 | 30 | -1.64 | 0.0455 |
| 116 | LOC102725228 | 0 | 19 | -1.64 | 0.0430 |
| 117 | FBLL1 | 4 | 39 | -1.64 | 0.0439 |
| 118 | LINC02837 | 1 | 22 | -1.64 | 0.0297 |
| 119 | TLN1 | 10 | 101 | -1.65 | 0.0057 |
| 120 | CACNG6 | 3 | 37 | -1.65 | 0.0293 |
| 121 | LOC124904647 | 2 | 28 | -1.65 | 0.0321 |
| 122 | PACRG-AS1 | 0 | 18 | -1.65 | 0.0386 |
| 123 | PRKACG | 2 | 86 | -1.65 | 0.0350 |
| 124 | LOC124903166 | 0 | 18 | -1.65 | 0.0420 |
| 125 | APOA4 | 0 | 18 | -1.65 | 0.0420 |
| 126 | LCN1 | 1 | 25 | -1.66 | 0.0430 |
| 127 | OR3A1 | 0 | 19 | -1.67 | 0.0347 |
| 128 | DUSP1 | 50 | 521 | -1.67 | 0.0003 |
| 129 | ROM1 | 3 | 35 | -1.68 | 0.0216 |
| 130 | ATP6AP1-DT | 4 | 54 | -1.68 | 0.0421 |
| 131 | FCER1G | 13 | 127 | -1.69 | 0.0017 |
| 132 | OR4F29 | 5 | 50 | -1.69 | 0.0185 |
| 133 | CYP4A11 | 1 | 23 | -1.69 | 0.0233 |
| 134 | LOC124904544 | 1 | 24 | -1.70 | 0.0252 |
| 135 | MIR4453HG | 2 | 35 | -1.70 | 0.0498 |
| 136 | LOC107986906 | 2 | 30 | -1.70 | 0.0226 |
| 137 | LOC101929305 | 2 | 30 | -1.70 | 0.0226 |
| 138 | LINC01800 | 0 | 19 | -1.70 | 0.0283 |
| 139 | SDHAF1 | 5 | 50 | -1.71 | 0.0255 |
| 140 | LOC124903187 | 2 | 34 | -1.71 | 0.0389 |
| 141 | TEX53 | 1 | 67 | -1.71 | 0.0456 |
| 142 | LOC107984421 | 3 | 36 | -1.71 | 0.0172 |
| 143 | HSPA7 | 9 | 121 | -1.72 | 0.0139 |
| 144 | LOC105375202 | 2 | 33 | -1.72 | 0.0313 |
| 145 | LOC107985038 | 0 | 20 | -1.72 | 0.0301 |
| 146 | PCOLCE | 4 | 56 | -1.72 | 0.0332 |
| 147 | LOC105370603 | 0 | 19 | -1.72 | 0.0280 |
| 148 | SNAI3 | 4 | 56 | -1.73 | 0.0313 |
| 149 | CDCP2 | 4 | 56 | -1.73 | 0.0313 |
| 150 | KLF18 | 0 | 19 | -1.73 | 0.0303 |
| 151 | SOWAHB | 1 | 24 | -1.73 | 0.0206 |
| 152 | MOCS2-DT | 2 | 35 | -1.73 | 0.0438 |
| 153 | CYP4F35P | 3 | 49 | -1.73 | 0.0476 |
| 154 | LOC124900652 | 1 | 25 | -1.73 | 0.0248 |
| 155 | TREML1 | 11 | 111 | -1.74 | 0.0056 |
| 156 | TRNI | 38 | 380 | -1.74 | 0.0150 |
| 157 | LOC105369183 | 3 | 42 | -1.74 | 0.0255 |
| 158 | LOC105371470 | 1 | 29 | -1.75 | 0.0462 |
| 159 | ZG16 | 2 | 30 | -1.75 | 0.0180 |
| 160 | BOLA1 | 6 | 82 | -1.75 | 0.0188 |
| 161 | LINC00336 | 2 | 37 | -1.76 | 0.0407 |
| 162 | SLITRK5 | 3 | 43 | -1.76 | 0.0248 |
| 163 | LOC101928502 | 0 | 20 | -1.76 | 0.0220 |
| 164 | HOXA13 | 3 | 39 | -1.76 | 0.0167 |
| 165 | FOLR2 | 1 | 24 | -1.77 | 0.0176 |
| 166 | BEX3 | 26 | 266 | -1.77 | 0.0020 |
| 167 | CD68 | 14 | 144 | -1.77 | 0.0014 |
| 168 | CALML3-AS1 | 1 | 27 | -1.77 | 0.0266 |
| 169 | KLHL40 | 2 | 34 | -1.78 | 0.0240 |
| 170 | SCARNA22 | 40 | 410 | -1.78 | 0.0230 |
| 171 | LINC01940 | 2 | 40 | -1.78 | 0.0492 |
| 172 | P2RX2 | 2 | 31 | -1.78 | 0.0168 |
| 173 | CD74 | 28 | 290 | -1.78 | 0.0008 |
| 174 | LOC105370980 | 11 | 195 | -1.78 | 0.0248 |
| 175 | LOC124902375 | 28 | 1524 | -1.78 | 0.0004 |
| 176 | RGN | 2 | 36 | -1.78 | 0.0292 |
| 177 | LINC02037 | 2 | 40 | -1.79 | 0.0435 |
| 178 | LOC101060187 | 2 | 38 | -1.79 | 0.0356 |
| 179 | LDHAL6B | 0 | 22 | -1.79 | 0.0384 |
| 180 | GP9 | 12 | 135 | -1.79 | 0.0018 |
| 181 | SUMO4 | 6 | 90 | -1.79 | 0.0256 |
| 182 | UBL4A | 10 | 154 | -1.79 | 0.0063 |
| 183 | AQP8 | 2 | 35 | -1.80 | 0.0235 |
| 184 | NXPH4 | 2 | 35 | -1.80 | 0.0234 |
| 185 | OTOR | 3 | 50 | -1.81 | 0.0344 |
| 186 | TRNS2 | 66 | 691 | -1.81 | 0.0173 |
| 187 | LINC00642 | 0 | 20 | -1.81 | 0.0254 |
| 188 | DDX11L10 | 7 | 103 | -1.81 | 0.0191 |
| 189 | LOC105372685 | 3 | 48 | -1.81 | 0.0270 |
| 190 | TNFSF9 | 3 | 50 | -1.81 | 0.0315 |
| 191 | PTPRT-AS1 | 1 | 26 | -1.82 | 0.0247 |
| 192 | TRNM | 156 | 2746 | -1.82 | 0.0003 |
| 193 | LOC124903198 | 0 | 20 | -1.82 | 0.0383 |
| 194 | LOC124902074 | 3 | 47 | -1.83 | 0.0226 |
| 195 | HCRT | 2 | 84 | -1.83 | 0.0122 |
| 196 | CFD | 1 | 25 | -1.83 | 0.0147 |
| 197 | CXCL5 | 12 | 129 | -1.83 | 0.0050 |
| 198 | PDIA2 | 2 | 41 | -1.84 | 0.0377 |
| 199 | SOX15 | 1 | 29 | -1.84 | 0.0290 |
| 200 | FAM131C | 2 | 37 | -1.84 | 0.0224 |
| 201 | CAMP | 10 | 109 | -1.85 | 0.0018 |
| 202 | TRNV | 126 | 1364 | -1.85 | 0.0032 |
| 203 | HCAR2 | 11 | 147 | -1.86 | 0.0168 |
| 204 | ZC3H18-AS1 | 1 | 27 | -1.86 | 0.0135 |
| 205 | LOC122455338 | 6 | 92 | -1.86 | 0.0252 |
| 206 | CTSG | 2 | 31 | -1.87 | 0.0142 |
| 207 | C1orf232 | 0 | 21 | -1.87 | 0.0158 |
| 208 | BHLHA15 | 2 | 40 | -1.88 | 0.0262 |
| 209 | LINC02151 | 0 | 24 | -1.88 | 0.0243 |
| 210 | LOC101060341 | 1 | 33 | -1.88 | 0.0320 |
| 211 | TRIM67-AS1 | 1 | 35 | -1.89 | 0.0415 |
| 212 | TP53AIP1 | 3 | 55 | -1.89 | 0.0284 |
| 213 | GAL3ST3 | 3 | 55 | -1.89 | 0.0284 |
| 214 | LOC105369358 | 0 | 23 | -1.89 | 0.0237 |
| 215 | LOC105376601 | 2 | 39 | -1.89 | 0.0216 |
| 216 | LOC124905202 | 2 | 42 | -1.90 | 0.0258 |
| 217 | LCN2 | 8 | 91 | -1.90 | 0.0023 |
| 218 | PTGER1 | 3 | 57 | -1.91 | 0.0342 |
| 219 | SRPK3 | 0 | 23 | -1.91 | 0.0150 |
| 220 | SPARC | 10 | 114 | -1.92 | 0.0029 |
| 221 | LRRC26 | 2 | 35 | -1.92 | 0.0131 |
| 222 | OR4D11 | 1 | 26 | -1.92 | 0.0268 |
| 223 | POTEKP | 1 | 33 | -1.93 | 0.0237 |
| 224 | ZNF528-AS1 | 3 | 56 | -1.93 | 0.0229 |
| 225 | KIF26A-DT | 1 | 30 | -1.93 | 0.0137 |
| 226 | CYP2A7 | 2 | 46 | -1.93 | 0.0304 |
| 227 | LINC00563 | 1 | 101 | -1.93 | 0.0110 |
| 228 | LINC03029 | 2 | 47 | -1.93 | 0.0482 |
| 229 | ADAMTSL5 | 3 | 49 | -1.94 | 0.0128 |
| 230 | LOC124901451 | 3 | 39 | -1.94 | 0.0374 |
| 231 | OR5C1 | 3 | 44 | -1.94 | 0.0147 |
| 232 | LOC105374353 | 0 | 25 | -1.95 | 0.0199 |
| 233 | LOC107984890 | 1 | 28 | -1.95 | 0.0082 |
| 234 | LOC101929413 | 1 | 31 | -1.95 | 0.0151 |
| 235 | PRR15 | 0 | 23 | -1.95 | 0.0129 |
| 236 | SHISA8 | 3 | 54 | -1.95 | 0.0199 |
| 237 | KCNK1 | 0 | 23 | -1.96 | 0.0093 |
| 238 | LOC124902405 | 8 | 94 | -1.96 | 0.0086 |
| 239 | KRT26 | 2 | 50 | -1.97 | 0.0381 |
| 240 | S100A11 | 16 | 190 | -1.98 | 0.0002 |
| 241 | LOC124907746 | 1 | 35 | -1.98 | 0.0243 |
| 242 | MUC15 | 0 | 24 | -1.98 | 0.0093 |
| 243 | FLNA | 15 | 208 | -1.99 | 0.0006 |
| 244 | G0S2 | 46 | 560 | -1.99 | 0.0063 |
| 245 | LOC105375107 | 1 | 41 | -2.00 | 0.0445 |
| 246 | TUBB1 | 12 | 150 | -2.00 | 0.0023 |
| 247 | SNORA104 | 19 | 227 | -2.00 | 0.0313 |
| 248 | S100A8 | 18 | 217 | -2.00 | 0.0003 |
| 249 | LOC105374672 | 0 | 30 | -2.00 | 0.0332 |
| 250 | TRNL1 | 258 | 3188 | -2.01 | 0.0001 |
| 251 | LOC124903767 | 3 | 41 | -2.01 | 0.0248 |
| 252 | ACTRT2 | 0 | 30 | -2.02 | 0.0362 |
| 253 | LOC124903397 | 1 | 37 | -2.03 | 0.0225 |
| 254 | LOC107985987 | 1 | 38 | -2.03 | 0.0218 |
| 255 | GPRIN1 | 2 | 44 | -2.03 | 0.0119 |
| 256 | CTSA | 11 | 147 | -2.04 | 0.0007 |
| 257 | TRNL2 | 96 | 1185 | -2.04 | 0.0007 |
| 258 | TMDD1 | 2 | 40 | -2.05 | 0.0270 |
| 259 | FCN1 | 15 | 1619 | -2.07 | 0.0001 |
| 260 | ATP8 | 8768 | 112230 | -2.08 | 0.0000 |
| 261 | LOC105375792 | 1 | 37 | -2.09 | 0.0121 |
| 262 | IFITM3 | 23 | 295 | -2.09 | 0.0001 |
| 263 | SRSF3P2 | 3 | 106 | -2.10 | 0.0088 |
| 264 | H2BC21 | 23 | 328 | -2.10 | 0.0002 |
| 265 | CAPN1-AS1 | 14 | 197 | -2.10 | 0.0003 |
| 266 | TRND | 137 | 1769 | -2.11 | 0.0011 |
| 267 | ANGPTL7 | 2 | 49 | -2.11 | 0.0108 |
| 268 | LOC124903014 | 2 | 53 | -2.11 | 0.0195 |
| 269 | PRODH2 | 1 | 130 | -2.12 | 0.0033 |
| 270 | LOC107986852 | 7 | 92 | -2.12 | 0.0082 |
| 271 | H2BC11 | 16 | 210 | -2.12 | 0.0015 |
| 272 | TRAV12-3 | 2 | 844 | -2.13 | 0.0128 |
| 273 | LOC124903734 | 6 | 1000 | -2.13 | 0.0034 |
| 274 | KIRREL3-AS3 | 0 | 33 | -2.14 | 0.0255 |
| 275 | GAS8-AS1 | 1 | 30 | -2.14 | 0.0347 |
| 276 | FERD3L | 0 | 33 | -2.15 | 0.0355 |
| 277 | RNASE2 | 5 | 67 | -2.15 | 0.0319 |
| 278 | TRR-TCG2-1 | 23 | 309 | -2.15 | 0.0360 |
| 279 | LINC01686 | 0 | 25 | -2.15 | 0.0184 |
| 280 | LOC124902211 | 0 | 32 | -2.16 | 0.0173 |
| 281 | IGLC2 | 14 | 190 | -2.17 | 0.0015 |
| 282 | TMSB4X | 342 | 4625 | -2.17 | 0.0001 |
| 283 | RGS2 | 17 | 248 | -2.18 | 0.0000 |
| 284 | H2AC11 | 6 | 107 | -2.21 | 0.0069 |
| 285 | GHET1 | 2 | 119 | -2.21 | 0.0044 |
| 286 | HSD3B7 | 2 | 54 | -2.22 | 0.0078 |
| 287 | CMTM5 | 10 | 202 | -2.22 | 0.0013 |
| 288 | HCFC1-AS1 | 2 | 50 | -2.23 | 0.0064 |
| 289 | H4C12 | 2 | 39 | -2.23 | 0.0329 |
| 290 | TAGLN2 | 30 | 446 | -2.23 | 0.0000 |
| 291 | ITGA2B | 11 | 157 | -2.23 | 0.0007 |
| 292 | TRNG | 40 | 565 | -2.23 | 0.0039 |
| 293 | LOC124905978 | 0 | 31 | -2.24 | 0.0050 |
| 294 | S100A12 | 21 | 299 | -2.24 | 0.0001 |
| 295 | OR4D5 | 1 | 50 | -2.24 | 0.0356 |
| 296 | PGRMC1 | 12 | 174 | -2.25 | 0.0003 |
| 297 | TRNR | 65 | 933 | -2.26 | 0.0005 |
| 298 | ND6 | 21682 | 318653 | -2.27 | 0.0000 |
| 299 | CRYBA2 | 0 | 40 | -2.27 | 0.0205 |
| 300 | LOC105374937 | 0 | 36 | -2.27 | 0.0159 |
| 301 | TONSL-AS1 | 0 | 38 | -2.27 | 0.0146 |
| 302 | LOC102724417 | 0 | 36 | -2.27 | 0.0103 |
| 303 | H2AC21 | 1 | 33 | -2.27 | 0.0147 |
| 304 | MYL9 | 11 | 174 | -2.28 | 0.0002 |
| 305 | C19orf73 | 1 | 149 | -2.29 | 0.0193 |
| 306 | SNORA109 | 5 | 74 | -2.30 | 0.0308 |
| 307 | ND2 | 71080 | 1057838 | -2.30 | 0.0000 |
| 308 | ATXN2-AS | 2 | 49 | -2.31 | 0.0274 |
| 309 | CEBPD | 19 | 291 | -2.32 | 0.0000 |
| 310 | IGLC3 | 6 | 91 | -2.33 | 0.0195 |
| 311 | DEFA3 | 10 | 152 | -2.34 | 0.0014 |
| 312 | PTGDR2 | 2 | 58 | -2.34 | 0.0044 |
| 313 | CLU | 10 | 164 | -2.34 | 0.0002 |
| 314 | CYTB | 37222 | 570270 | -2.34 | 0.0000 |
| 315 | H3C10 | 50 | 779 | -2.35 | 0.0001 |
| 316 | ND5 | 22348 | 355417 | -2.38 | 0.0000 |
| 317 | GPR25 | 0 | 205 | -2.39 | 0.0039 |
| 318 | ND4 | 62968 | 993460 | -2.39 | 0.0000 |
| 319 | ND1 | 79732 | 1258215 | -2.39 | 0.0000 |
| 320 | ATP6 | 47624 | 761651 | -2.41 | 0.0000 |
| 321 | LOC107987244 | 1 | 700 | -2.46 | 0.0075 |
| 322 | FCGR3B | 12 | 216 | -2.46 | 0.0000 |
| 323 | CLDN5 | 7 | 122 | -2.46 | 0.0003 |
| 324 | GNG11 | 14 | 249 | -2.47 | 0.0001 |
| 325 | COX1 | 96594 | 1648342 | -2.50 | 0.0000 |
| 326 | COX3 | 39481 | 681256 | -2.52 | 0.0000 |
| 327 | IFITM2 | 90 | 1568 | -2.53 | 0.0000 |
| 328 | MIR12136 | 20189 | 421677 | -2.55 | 0.0000 |
| 329 | COX2 | 65500 | 1157833 | -2.55 | 0.0000 |
| 330 | SMPD5 | 0 | 33 | -2.56 | 0.0028 |
| 331 | H3Y1 | 4 | 568 | -2.57 | 0.0013 |
| 332 | TRNP | 33520 | 607916 | -2.58 | 0.0000 |
| 333 | H2AC6 | 79 | 1449 | -2.60 | 0.0000 |
| 334 | ND3 | 30775 | 580743 | -2.65 | 0.0000 |
| 335 | TRNF | 193 | 3745 | -2.69 | 0.0000 |
| 336 | NRGN | 57 | 1107 | -2.69 | 0.0000 |
| 337 | CAVIN2 | 13 | 262 | -2.71 | 0.0000 |
| 338 | CXCR2P1 | 13 | 257 | -2.72 | 0.0001 |
| 339 | MIR4485 | 270 | 5359 | -2.73 | 0.0000 |
| 340 | RNR2 | 222856 | 4434046 | -2.73 | 0.0000 |
| 341 | PPBP | 134 | 2738 | -2.77 | 0.0000 |
| 342 | ND4L | 9233 | 190961 | -2.77 | 0.0000 |
| 343 | OR13C8 | 11 | 2249 | -2.88 | 0.0002 |
| 344 | S100A9 | 109 | 2412 | -2.88 | 0.0000 |
| 345 | RNR1 | 107197 | 2433831 | -2.92 | 0.0000 |
| 346 | TRNE | 35 | 814 | -2.96 | 0.0006 |
| 347 | LOC124901863 | 4 | 96 | -2.96 | 0.0316 |
| 348 | PF4 | 160 | 3947 | -3.04 | 0.0000 |
| 349 | LOC124903595 | 2 | 132 | -3.19 | 0.0288 |
| 350 | PF4V1 | 13 | 368 | -3.19 | 0.0000 |
| 351 | MIR573 | 0 | 51 | -3.20 | 0.0495 |
| 352 | TRNC | 159 | 6113 | -3.43 | 0.0000 |
| 353 | MIR521-2 | 2 | 90 | -3.44 | 0.0425 |
| 354 | TRU-TCA1-1 | 3 | 115 | -3.51 | 0.0264 |
| 355 | TRP-TGG3-3 | 12 | 857 | -3.52 | 0.0468 |
| 356 | LOC124906355 | 20 | 144 | -3.54 | 0.0452 |
| 357 | LOC124903910 | 2 | 165 | -3.58 | 0.0024 |
| 358 | TRNY | 39 | 2658 | -3.59 | 0.0000 |
| 359 | MIR518E | 0 | 72 | -3.70 | 0.0116 |
| 360 | SNORD35B | 0 | 74 | -3.74 | 0.0107 |
| 361 | MIR524 | 0 | 77 | -3.79 | 0.0488 |
| 362 | LOC124900530 | 0 | 78 | -3.81 | 0.0210 |
| 363 | TRE-CTC1-1 | 5 | 211 | -3.81 | 0.0244 |
| 364 | LOC124900325 | 0 | 87 | -3.97 | 0.0151 |
| 365 | TRS-TGA1-1 | 4 | 211 | -4.09 | 0.0049 |
| 366 | TRK-CTT1-2 | 2 | 150 | -4.18 | 0.0147 |
| 367 | MIR4322 | 2 | 2291 | -4.62 | 0.0009 |
| 368 | LOC124900196 | 38 | 217 | -4.70 | 0.0062 |
| 369 | MIR518D | 42 | 235 | -4.82 | 0.0121 |
| 370 | MIR4675 | 0 | 171 | -4.94 | 0.0113 |
